# Cellular Communication through Light

**DOI:** 10.1371/journal.pone.0005086

**Published:** 2009-04-01

**Authors:** Daniel Fels

**Affiliations:** 1 Swiss Tropical Institute (STI), Basel, Switzerland; 2 UPMC Univ. Paris 06, Laboratoire de Parasitologie Evolutive - UMR 7103, Paris, France; University of Sydney, Australia

## Abstract

Information transfer is a fundamental of life. A few studies have reported that cells use photons (from an endogenous source) as information carriers. This study finds that cells can have an influence on other cells even when separated with a glass barrier, thereby disabling molecule diffusion through the cell-containing medium. As there is still very little known about the potential of photons for intercellular communication this study is designed to test for non-molecule-based triggering of two fundamental properties of life: cell division and energy uptake. The study was performed with a cellular organism, the ciliate *Paramecium caudatum*. Mutual exposure of cell populations occurred under conditions of darkness and separation with cuvettes (vials) allowing photon but not molecule transfer. The cell populations were separated either with glass allowing photon transmission from 340 nm to longer waves, or quartz being transmittable from 150 nm, i.e. from UV-light to longer waves. Even through glass, the cells affected cell division and energy uptake in neighboring cell populations. Depending on the cuvette material and the number of cells involved, these effects were positive or negative. Also, while paired populations with lower growth rates grew uncorrelated, growth of the better growing populations was correlated. As there were significant differences when separating the populations with glass or quartz, it is suggested that the cell populations use two (or more) frequencies for cellular information transfer, which influences at least energy uptake, cell division rate and growth correlation. Altogether the study strongly supports a cellular communication system, which is different from a molecule-receptor-based system and hints that photon-triggering is a fine tuning principle in cell chemistry.

## Introduction

Information transfer is a life principle. On a cellular level we generally assume that molecules are carriers of information, yet there is evidence for non-molecular information transfer due to endogenous coherent light [1]. This light is ultra-weak, is emitted by many organisms [2]–[5], including humans [6], [7] and is conventionally described as biophoton emission [8]–[10]. Research on biophotons focuses mainly on the physical aspects and origin [11]–[13], non-invasive diagnostics [14], [15], and emission during meiosis [16] or embryogenesis [17], [18]. Some organisms, e.g. the crustacean *Daphnia magna* [19] absorb biophotons from their neighbours - so called *photon sucking* [20] – and the uptake can differ among classes of individuals, e.g. healthy as compared to malign cells [21]. Although biophotons may carry biologically relevant information [12], [13], [22], only very little is known about whether individuals indeed use them for sending and receiving information. A few studies (with populations separated from each other molecularly but not electromagnetically) strongly suggest biophotons as transmitters of information: e.g., onion roots influence mitosis positively in neighbouring onion roots (supposedly due to so-called *mitogenetic radiation* [23], being probably effective in the UV-range [24]); yeast cells, which emit biophotons in the UV- and the visible range [25], affect growth in other yeast cells positively [26]; tissue cells arrange themselves in a non-random manner according to the pattern of tissue cells on the opposite side of a glass slide [27]; and germinating *Fucus*-zygotes probably sense biophotons emitted by their living substrate to which they direct their growth [28].

It was the paucity of more detailed knowledge on biophotons as a means for electromagnetic information transmission that motivated this study - to examine whether cell populations of the ciliate *Paramecium caudatum* that were separated from each other by a glass or quartz barrier would mutually affect cell division (growth) or vacuole formation (i.e. energy uptake) in a neighbouring population. *Paramecia* are a good model organism for this study because they are maintained easily in the lab and the traits relevant to this study, i.e. population growth and feeding, can easily be assessed. Glass and quartz cuvettes having different transmission spectra were used a) to separate populations molecularly but not electromagnetically from each other and b) to obtain information on the triggering frequencies, specifically whether they were due to UV-light or waves that are longer than UV-light.

In a series of experiments, population growth and the feeding rate of *Paramecium caudatum* depended significantly on (i) the presence or absence of a neighbouring population, (ii) the number of cells in the neighbouring population and (iii) the material (glass or quartz) separating these populations. The results strongly support the existence of a non-molecular information-carrying system that is based on photons.

## Materials and Methods

### Study organism

All experiments were performed with the ciliate *Paramecium caudatum*. Its cultures were kept at a density of about 100 individuals/ml in Erlenmeyer beakers (300 ml) at 23–25°C and fed every 2–3 days with a medium containing the bacteria *Serratia marcenscens* and particles of dried salad (the food source of the bacteria). Under a binocular microscope at 30-fold magnification, the ciliates (length about 0.3 mm) can easily be counted or picked up with a micropipette. For picking and counting 1 ml of the culture was distributed into four flat sections of a glass well.

### Cuvettes

**Figure 1:**
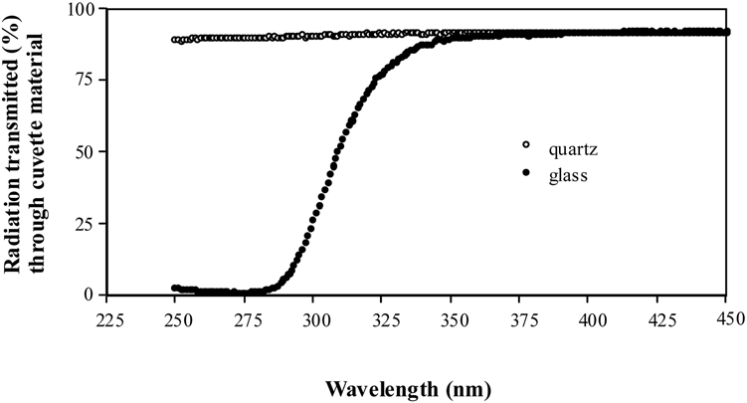
Transmission of electromagnetic waves through 1.5 mm of cuvette material. The graph shows the wavelengths where transmission through glass differs from transmission through quartz.

Populations of *Paramecia* were separated from each other with cuvettes with a wall thickness of 1.5 mm. Two sizes of cuvettes were used: 2.3 mm×2.3 mm×40 mm, and 1.5 mm×1.5 mm×45 mm. The cuvettes were made of the inert materials glass and quartz. Although both consist of SiO_2_, glass is amorphous and quartz crystalline. Furthermore, quartz cuvettes transmit radiation with wavelengths greater than 150 nm (the quartz was verified at the Institute of Physics, University of Basel, Switzerland, and transmission was measured down to 250 nm, where it was still 90%; Fig. 1). The glass was very pure, i.e. mainly SiO_2_, allowing high transmission of light down to 340 nm (measured at the Institute of Physics, University of Basel) (see Fig. 1), hence, the glass cuvettes served as UV-filters (strictly speaking the glass used in this study allowed only the transmission of weak UV-light). Figure 1 shows how transmission through glass decreases between 340 nm and 280 nm, and rapidly from 90% to 0% with 50% transmission at about 310 nm. Quartz still transmits 90% of waves with a length of 250 nm. Note that transmission of both quartz and glass remained at 90% for all wavelengths measured up to 2500 nm (not shown in the graph).

### Experimental set-up

**Figure 2:**
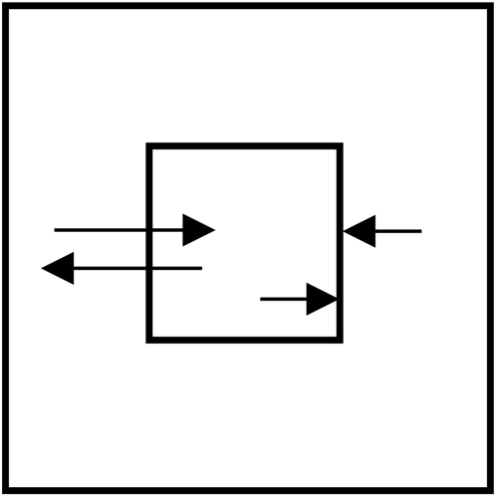
Vertical section of an experimental unit of small and large cuvettes. Arrows pointing at the inner cuvette show the molecule barrier. Arrows going through the inner cuvette refer to photon transmission.

I performed three major experiments to test – indirectly – for effects of endogenous light on the growth and feeding rate of *Paramecium* populations. The basic setup consisted of a small cuvette placed within a larger one, further ascribed as a unit (see Fig. 2). Each cuvette contained 1 ml of medium and a given number of *Paramecia*. The units disabled molecular but allowed electromagnetic interactions between two populations of *Paramecia*.

In the first experiment (1a) mutual influence between the two separated populations was tested, i.e. both populations were considered as sender and receiver. In the second experiment (2), inner populations were taken as senders and their effect on the outer receiving populations measured. In the third experiment, outer populations were taken as senders and their effect on inner receiving populations were measured (refer to the summary table: Table 1).

**Table 1:** Summary of the major results.

Exp*	Testing on	Cells in∶out	Sender>receiver	Material	Effects
1a	Cell division	5∶100	Inside>outside	Glass	Increase**
		5∶100	Inside>outside	Quartz	No
		5∶100	Inside<outside	Glass	No
		5∶100	Inside<outside	Quartz	Decrease**
2	Cell division	5∶25	Inside>outside	Glass	No
		5∶25	Inside>outside	Quartz	Decrease**
3	Energy uptake	15(20)∶15(20)	Inside<outside	Glass	Decrease
		15(20)∶300(400)	Inside<outside	Glass	Increase
		15(20)∶15(20)	Inside<outside	Quartz	Increase
		15(20)∶300(400)	Inside<outside	Quartz	Decrease

The effects on receiver populations depend on the number of receiving and/or sender cells in the inner and outer cuvette (the so called units) as well as on the separating material (quartz or glass). In experiment 1a and 2 cell division (growth) was assessed 48 hrs after the mutual exposure of cell populations. In experiment 3 energy uptake (vacuole formation) of cells was assessed 3 hrs after the mutual exposure.

**Figure 3:**
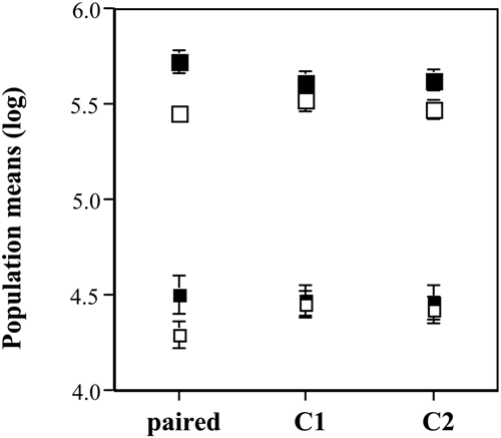
Mutual effects on growth between optically coupled cell populations. The graph shows in the upper row the sizes of the larger outer populations and in the lower row those of the smaller inner populations. The filled squares refer to treatments using glass cuvettes, the open squares to those with quartz cuvettes. The x-axis shows the three treatment groups: paired are the combined (outer and inner) populations; C1 refers to the controls using medium instead of a second population and C2 is the control with demineralised water.

**Figure 4:**
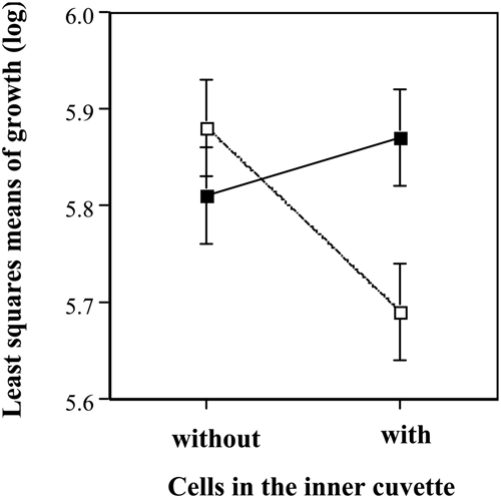
Effects from cells in the inner cuvette to cells in the outer cuvette. The graph shows the combined effects of material and presence or absence of cells in the inner cuvette on cell growth in the outer population. Filled squares refer to separation by glass and open squares to separation by quartz.

* For experiment 1a see also figure 3, tables 2 and 3; for exp. 2 see figure 4 and table 4; for exp. 3 see figure 6 and table 5.

** The significance of these effects follows from a *contrast analysis*, which is described in the text.

#### Experiment 1a

The aim of this experiment was to measure the effect of dense populations (initial size: 100 cells) in the outer cuvettes on the growth of small populations (initial size: 5 cells) in the inner cuvettes and *vice versa*. Individual *Paramecium* were added to the corresponding cuvette containing 1 ml of medium. The effects were compared for units of glass and quartz cuvettes. In order to test for mutual effects of the populations through glass, growth of non-mutually exposed populations was assessed for comparison. There were two types of such controls: one with cuvettes that contained no *Paramecium* but 1 ml medium only. This type controlled for presence and absence of Paramecia. In the second type of control the cuvettes again contained no *Paramecia* but 1 ml demineralised water. With this control, effects coming from the medium alone were tested. Each experimental block consisted finally of 10 units: 2 types of material (glass or quartz) ×5 combinations of *Paramecium* (an inner cuvette containing 5 *Paramecium* within a cuvette containing 100 *Paramecium*, an inner cuvette with 5 *Paramecium* within a cuvette containing medium only or demineralised water, and an inner cuvette containing medium only or demineralised water within a cuvette containing 100 *Paramecium*).

Twenty-eight blocks were assayed in 14 experimental sessions that were performed at different days. In each session two blocks were randomly placed on a four by five grid, where the units were optically separated from each other by a black carton. The grid itself was placed in a cardboard box. The populations were kept for 48 hours at constant room temperature, which was of 27°C in 10 sessions, 25°C, in one session, 23°C in one session and 22°C in two sessions. During the 48 hour period of growth a second cardboard box covered the one with the grid and was wrapped with a double-layered sheet of black cloth. This prevented external light from influencing the populations.

After 48 hours, each population was distributed into four sections of a glass well. The number of individuals per population was assessed with a hand counter. The mean of two or, if the difference between two counts was high, of three counts served as data points.

#### Experiment 1b

A potential problem with experiment 1a was that *Paramecium* might change the culture medium, so that the difference between the control (medium only) and the cuvettes containing *Paramecium* could reflect differences in the medium. In this experiment, I therefore assayed a possible difference between culture medium and fresh medium (the one added regularly to the cultures as described above). The culture medium was obtained by filtering a culture once through a gauze into a 50 ml tube. The *Paramecium* then tend to swim to the ground of the tube. The remaining medium in the upper part was taken and checked for *Paramecium*. One ml of the culture medium or fresh medium (neither one containing cells) was then placed into a large cuvette. Small cuvettes containing 5 individuals were then placed into these large cuvettes. An experimental block thus contained 4 types of units: glass or quartz outer cuvettes containing fresh or culture medium (the inner cuvettes were of the same material than the outer ones; either glass or quartz). Blocks were replicated 5 times per experimental session on a 4×5 grid (see experiment 1a) and four such sessions were performed at different days.

#### Experiment 2

This experiment assayed the effect of a small inner population on the growth of an outer population that was smaller than in experiment 1 (25 individuals). It tested, furthermore, for all possible combinations of the two cuvette materials. A block consisted, therefore, of 8 units: the inner cuvette made of glass or quartz (two possibilities), containing no or 5 cells (2 possibilities), combined with the outer glass or quartz cuvette (two possibilities). An experimental session consisted of two such blocks (i.e. 16 units in the grid) and was repeated 5 times at different days. The exposure period (under conditions as in experiment 1) was 48 hours before counting the number of individuals from each of the cuvettes.

#### Experiment 3

Rather than considering the growth of a population, this experiment assayed the feeding rate of individuals, assessed as the number of vacuoles within an individual's cytoplasm. This demands the fixation of individual cells, a method described elsewhere [29]. In a preliminary experiment latex beads were used to test for a relationship between vacuole number and feeding effort; using latex beads is a well-established method to quantify feeding behaviour of protozoa [30]. 200 ciliates were kept during 2 hours in 1 ml medium in an Eppendorfer tube. Round polystyrene latexbeads (SIGMA; LB30-2ML; mean particle size: 3 µm) were added at a concentration of 30,000 beads/1 ml. The ciliates had ingested latexbeads and a significant correlation between number of vacuoles and number of latexbeads was found (n = 51; coefficient of correlation = 0.40; p<0.0035**). Since vacuoles contain bacteria, the number of vacuoles can be used as a direct measure of energy uptake.

The main experiment is methodologically distinct from experiments 1 and 2 in two ways. First, it has a blind (random) design and second, the cells were not individually picked but population densities were assessed and fractions taken to obtain the desired cell densities. The effect of outer populations on inner populations was tested.

Two experiments were performed. In the first experiment 15 cells were in the inner cuvette and 15 or 300 cells in the outer cuvette. Furthermore, the material was for each unit the same, both inner and outer cuvette were either of glass or of quartz. This led to 4 units each of which was replicated four times within the experiment. The experimental block consisted, therefore in 16 units that were placed in the grid (as mentioned above). The second experiment differed from the first one slightly in cell numbers: in the inner cuvette there were always 20 cells, while there were 20 or 400 cells in the outer cuvette. Otherwise everything was kept as in the first experiment.

In both experiments the mutual exposure lasted for 3 hours. During exposure the paired populations were kept in a box as in experiments 1 and 2 on growth. When taking out the ciliates for fixation, the box was repeatedly opened and closed. Consequently, the later a unit was taken out of the box the more this unit would experience light from the laboratory illumination (In the second experiment this exposure to external light was kept at a minimum: the box was opened in a dark room with a few standby-lights of incubators only). For the analysis, however, the four replicates per treatment group were separated into the first two replicates (of each treatment that were taken from the random distribution in the box) and the last two replicates.

### Analysis

All experiments were tested with an ANOVA (or t-test for contrast analysis in experiments 1 and 2) using the statistical package JMP [31]. All analysis was done on log-transformed data of population sizes and vacuole numbers, respectively.

## Results

The main results of effects of neighboring cell populations through glass are summarized in a table (Table 1).

### Experiment 1a

Both populations, the smaller in the inner cuvette and the larger in the outer cuvette, experienced strong effects from the day(s) of experimentation and from the cuvette material as well as from interactions between the two. More importantly, both day(s) of experimentation and also material interacted significantly with the mutual exposure of cell populations, i.e. the treatment (Table 2 and 3, Fig. 3). Analysing the material×treatment interaction, I contrasted paired versus non-paired (control) populations. As there were significant effects for both large and small populations (statistics not shown), a separate test for effects due to the separating material was performed. This test showed, on the one hand, that large populations grew significantly better (than controls) when separated with glass from the small neighbour populations (t-test: degree of freedom = 2,84; sum of squares = 0.192; t-ratio = 2.736; p>t = 0.008***) but they grew as well as the controls when separated with quartz from the smaller neighbour populations (t-test: degree of freedom = 2,84; sum of squares = 0.042; t-ratio = −1.278; p>t = 0.205) (Fig. 3). On the other hand, the small populations grew as well as the controls when separated with glass from the large neighbour populations (t-test: degree of freedom = 2,84; sum of squares = 0.024; t-ratio = 0.742; p>t = 0.460) but they grew significantly worse (than controls) when separated with quartz from the large neighbouring populations (t-test: degree of freedom = 2,84; sum of squares = 0.366; t-ratio = −2.877; p>t = 0.005***) (Fig. 3).

**Table 2:** Effects on large populations in the outer cuvettes.

Source	DF	SS	F-ratio	P>F
Day effects (day)	13	7.495	22.511	<0.0001****
Material (mat)	1	1.291	50.389	<0.0001****
Day×mat	13	1.615	4.849	<0.0001****
Treatment (treat) *	2	0.036	0.708	0.496
Day×treat	26	1.154	1.733	0.032*
Mat×treat °	2	0.228	4.447	0.015*
Day×mat×treat	26	0.948	1.424	0.116

(ANOVA: DF = degree(s) of freedom; SS = sum of squares).

* Treatment refers to the presence or absence of a neighbouring population.

° The important result is the interaction of treatment with separating material.

#### Material effects

The (large) populations in the outer cuvette grew significantly better in glass than in quartz (independent from neighbours; statistics not shown; confer Fig. 3). The (small) populations in the inner cuvettes showed no effect due to cuvette material (statistics not shown): the differences (confer Table 3 and Fig. 3) are fully explained by the reduced growth of small populations when separated by quartz from their neighbour population (see contrast analysis above,).

**Table 3:** Effects on small populations in the inner cuvettes.

Source	DF	SS	F-ratio	P>F
Day effects (day)	13	21.735	37.827	<0.0001****
Material (mat)	1	0.323	7.300	0.008***
Day×mat	13	1.296	2.255	0.014*
Treatment (treat) *	2	0.113	1.282	0.283
Day×treat	26	1.151	1.001	0.048*
Mat×treat °	2	0.292	3.303	0.042*
Day×mat×treat	26	1.431	1.245	0.225

(ANOVA: DF = degree(s) of freedom; SS = sum of squares).

* Treatment refers to the presence or absence of a neighbouring population.

° The important result is the interaction of treatment with separating material.

An *a posteriori analysis* revealed that the better growing paired (small and large) populations in glass grew significantly correlated (ANOVA (linear fit): degree of freedom = 1; r^2^ = 0.21; F-ratio = 6.90; p>F = 0.0143) while the reduced growth of paired populations in quartz was not correlated (ANOVA (linear fit): degree of freedom = 1; r^2^ = 0.06; F-ratio = 1.52; p>F = 0.228).

### Experiment 1b

Testing for effects of fresh medium *versus* culture medium on cells that were growing in the inner cuvette revealed no effect (ANOVA: DF = 1; SS = 0.000; F-ratio = 0.001; p>F = 0.9755). There were no effects of material nor of material×medium interaction (statistics no shown).

### Experiment 2

This experiment on the effects of material and of the presence of cells in the inner cuvette on growth of cells in the outer cuvette revealed very strong material effects. When the outer cuvettes were of glass, growth of the outer population was significantly better than compared to outer populations in quartz cuvettes. The material of the inner cuvette did not affect this growth of the outer populations and there was no interaction between the materials of the inner and outer cuvettes. Also, the presence of cells in the inner cuvette (irrespective of material) did not affect growth either (Table 4). Only the interaction between inner and outer material and the presence or absence of cells showed a marginal effect on growth (Table 4). However, when combining the presence of the cells in the inner cuvette with the material that separates them from the outer populations, there was a significant interaction (Table 4). A contrast analysis showed that all combinations grew similarly (i.e. statistically indistinguishable) except for the paired populations that were separated by quartz: in these units the outer populations showed a significantly reduced growth (contrast analysis: degree of freedom = 1,68; F-ratio = 8.125; p>F = 0.0058***) (Fig. 4).

**Table 4:** Effects of cell and material combinations on outer populations.

Source	DF	SS	F-ratio	p>F
Day effects	4	18.73	90.27	>0.0001****
Material of cuvette outside (mat-out)	1	0.76	14.74	0.0003***
Material of cuvette inside (mat-in)	1	0.06	1.16	0.2848
Cells inside (cells-in)	1	0.11	2.03	0.1591
Mat-out×mat-in	1	0.08	1.60	0.2105
Mat-out×cells-in	1	0.01	0.16	0.6861
Mat-in×cells-in °	1	0.31	5.93	0.0175*
Mat-out×mat-in×cells-in	1	0.17	3.22	0.0773

(ANOVA: DF = degree(s) of freedom; SS = sum of squares).

° The important result is the interaction of material with (presence or absence of) cells.

**Figure 5:**
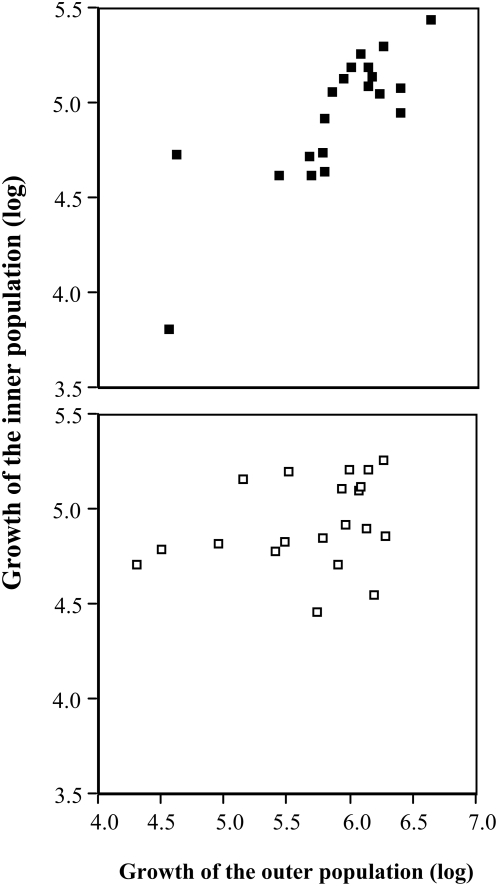
The coupling of growth between outer and inner population. The upper graph represents the significantly better growing paired populations separated by glass while the lower graph refers to the reduced growth of paired populations separated by quartz (compare fig 4, right half).

An *a posteriori analysis* of growth inside with growth outside showed that the better growing paired populations (separated by glass) were highly significantly correlated (ANOVA (linear fit): degree of freedom = 1; r^2^ = 0.67; F-ratio = 36.39; p>F = 0.0001) while the worse growing paired populations (separated by quartz) were not (ANOVA (linear fit): degree of freedom = 1; r^2^ = 0.07; F-ratio = 1.44; p>F = 0.245) (Fig. 5).

### Experiment 3

**Figure 6:**
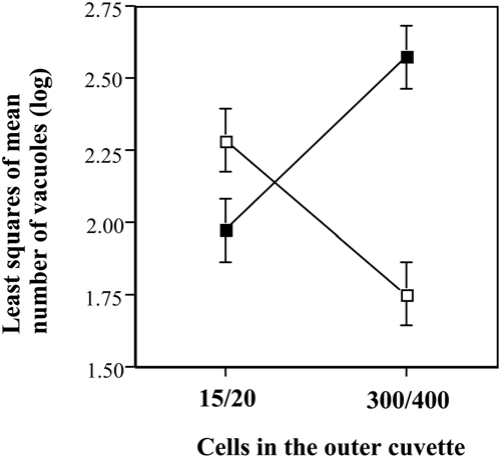
Effects of neighbouring cells on vacuole formation (energy uptake). The population size in the outer cuvette reflected either the size of the population in the inner cuvette (15 or 20) or a 20-fold of it (300 or 400). Filled squares refer to separation with glass, open squares to separation with quartz.

Pooling the two experiments and taking the subset of the first two replicates (i.e., those taken first out of the black box), it turned out that the number of vacuoles produced by the ciliates was significantly higher for the ciliates in glass cuvettes. There was no overall treatment effect. However, the interaction of material and treatment showed a highly significant effect on vacuole formation (Table 5, Fig. 6). When separated by quartz from a few neighbouring cells (15–20), vacuole formation was higher than for the glass units, but when separated from many neighbours (300–400 cells) it was the lowest of all treatments. The opposite effect was found for populations separated by glass (confer Fig. 6) The subset of the last two replicates showed neither main effects nor significant interactions (statistics not shown).

**Table 5:** Effects of outer populations on the number of vacuoles produced by inner populations.

Source	DF	SS	F-ratio	p>F
Material	1	2.078	5.475	0.021*
Treatment *	1	0.039	0.104	0.748
Material×treatment °	1	10.357	27.284	<0.0001****

(ANOVA: DF = degree(s) of freedom; SS = sum of squares).

* Treatment refers to many or few cells in the outer cuvette.

° The important result is the interaction of material with cell-number outside.

## Discussion

In the present study, three major experiments confirmed that separated populations of the ciliate *Paramecium caudatum* interact with each other through glass under conditions of complete darkness. A careful control showed that the interactions are due to conspecific cells and not to the medium containing bacteria. The mutual influence between the ciliates was found for cell division, growth correlation and energy uptake (vacuole formation). The general picture is that fundamental life properties such as cell division and energy uptake can be enhanced as well as reduced by a neighbouring population. Two factors influence this: the number of cells in the neighbouring population and the separating material.

Comparing these results with corresponding studies on onion roots [24], yeast cells [26], tissue cells [27] and zygote-germination [28] a major common feature appears: organisms (or isolated cells) can transmit information without the use of a molecular information carrier. The observed induction on growth, furthermore, hints at a universal property of growth regulation. However, this study gives a much more differentiated picture. First, cells can not only induce an increase in growth, but also a decrease. Second, although population sizes differed up to 20-fold between mutually exposed populations, influence was observed in both directions, namely large populations influencing small ones and *vice versa*; and interestingly, this depended on the separating material. Third, populations that experienced a positive or no induction on growth from their neighbours grew in correlation with these neighbours, while those populations growing more slowly due to neighbours showed no correlated growth with them. Fourth, the congruent results of the qualitatively comparable experiments 2 and 3 on growth and vacuole formation, respectively, suggest that the effects on growth resulted from a cumulative effect acting on feeding rate. Even though this does not exclude that the mutual cell-to-cell influence triggers cell division directly, energy uptake is, nonetheless a *conditio sine qua non* for cell division. Altogether, the results hint at a complex information system regulating cell growth, growth correlation and energy uptake.

Are these interactions based on biophotons? Clearly this was not measured and to my knowledge is not currently measurable, for the electromagnetic spatial dimension of cells is so far not reachable either for precise assessment or application. But there is strong indirect evidence for biophotons as information carriers coming from the separation of cell populations through the use of quartz or glass (a filter for UV-light below 340 nm, see above), which produces different results. If only frequencies above 340 nm were used, we should expect to find no differences between populations separated by glass or quartz, because both materials allow the transmission of these frequencies. Yet there were such differences. Likewise, if only frequencies below 340 nm were to be used, an effect on growth for populations separated by glass should not be observed, but there were effects. Consequently, one can deduce that at least two frequencies are in use, one above 340 nm and the other one below 340 nm (note that below 340 nm, transmission through glass can still occur but in decreased percentages as shown in Fig. 1). Furthermore, as separation with quartz can enhance as well as reduce growth and/or vacuole formation, either several frequencies (with opposite effects) are used below 340 nm or only one frequency is used, but it is modulated: The same argument goes for separation with glass. As the size of both stimulus populations and receiving populations has effects in either direction it may be that frequencies – or alternately the modulation of frequencies – depend, on the one hand, on the number of cells, i.e. the number of electromagnetic fields [1], [32] and on the other hand, are due to the (typically) non-linear behaviour of electromagnetic interactions in biological systems [1], [19], [22].

There was a material effect on the outer populations with glass resulting in better growth or energy uptake as compared to quartz. It is not within the scope of this study to address why this was the case. More important, however, is the fact that these pure material effects do not explain the neighbourhood effects, for they were repeatedly opposed to each other.

The evidence for electromagnetic information transfer is strong, and it is difficult to think of alternative mechanisms that could have produced similar results. One possibility is that molecules in a gaseous state left the cuvettes and influenced neighbouring populations. But this appears improbable because these molecules would have to diffuse only into one neighbouring cuvette (i.e. the right one). Note that for an inner cuvette the outer cuvette is indeed the nearest one, while for an outer cuvette, the nearest cuvette is the one standing next in the grid, i.e., the larger cuvette of another unit. This is so because of the differing heights of the smaller inner cuvette (45 mm) and the larger outer cuvette (40 mm). Furthermore, due to the random design, the treatments differ between neighbouring units in the grid. In addition, such molecules would have to be specific to the material of the cuvette and to the number of cells they originated from. If they existed, however, they would most probably produce a mixture in the microclimate (recall that the mutual exposure lasted for 48 hrs in exp. 1a and 2) that would be more or less the same for all cuvettes into which they might diffuse, and consequently lead to results that are independent from the treatments. As the results were very distinct between treatment groups, it is unlikely that such molecules were present.

Another alternative explanation for effects from the neighbouring cells would be heat production, i.e. infrared waves caused by cell metabolism. Once again however, there is the problem of omni-directional results: cells in the neighbouring cuvette would experience both enhancing and reducing effects on growth. Furthermore, the cells were exposed at 27°C and it seems doubtful that the small cells could heat up the water in the neighbouring cuvette due to metabolism. Finally, within the single frequency (above 340 nm) the results should not show differences between quartz and glass cuvettes (see argument above), yet, they do.

The goal of this study was to look for the potential of endogenous photons to act as triggering signals: under the conditions of the experiment an information transfer was indeed discerned. It is very probably due to photons emitted by cells, hence biophotons. Since the cells can communicate between populations separated by glass as described in this study, one may deduce that the cells do also communicate within a population, i.e. between cells. Cells, in addition to being a world of molecules, are also a world of electromagnetic fields that play major roles in morphogenesis of multicellular organisms [32]. Morphogenesis is a consequence of cell differentiation and cell migration, which first of all demands cell division. If the effects on cell division (growth) found in this and other studies reveal a common feature, we are obliged to accept that cells are not only a world of effective molecules but also a world of effective light. Interestingly, this is not really new: the electron transfer in chemical reactions (or within a molecule) is triggered by units of energy, the *quanta*. A photon is such a *quantum* and the electromagnetic fields of cells are assumed to be standing (coherent) waves that absorb and emit *quanta*, i.e. photons [1]. Biophoton interaction is understood as part of a communication system based on electromagnetic fields within and between cells [1], [32].

### Conclusions

Cells can influence each other without using a molecular signal for the purpose: this means that not all cellular processes are necessarily based on a molecule-receptor recognition. The non-molecular signals are most probably photons. If so, cells use more than one frequency for information transfer and mutual influence. The effects are manifold, acting positively or negatively on cell growth, correlated growth and energy uptake. Since there are already existing reports of the induction of chemical reactions through glass [33], [34], it might be that many cell processes are triggered by photons. Biophoton research is a non-invasive method that can give us valuable insights into non-molecular regulation of the life processes. If we can devote significant effort into this area of research we may one day develop non-invasive application technology with a fundamental impact into the nature of healthcare and medicine.
