# Correction: Cellular Communication through Light

**DOI:** 10.1371/annotation/8d99ccc5-cc76-44f4-b468-d63e42e0b9e1

**Published:** 2009-07-21

**Authors:** Daniel Fels

In the Materials and Methods subsection "Cuvettes" there is a typographical error in the cuvette dimensions. The correct measurements should be: 23 mm x 23 mm x 40 mm, and 15 mm x 15 mm x 45 mm.

